# Forty years on: a brief history of *clinical and experimental metastasis*

**DOI:** 10.1007/s10585-024-10281-7

**Published:** 2024-03-23

**Authors:** Jonathan P. Sleeman, Jörg Haier

**Affiliations:** 1 Medizinische Fakultät Mannheim, Universität Heidelberg European Center for Angioscience (ECAS), Ludolf-Krehl-Str. 13 - 17, D-68167 Mannheim, Germany; 2 Karlsruhe Institute for Technology (KIT), IBCS-BIP, Hermann-von-Helmholtz-Platz 1, 76344 Eggenstein- Leopoldshafen, Germany; 3 Comprehensive Cancer Center Hannover, Hannover Medical School, Hannover, Germany

The first issue of *Clinical & Experimental Metastasis* was published in March 1983. To mark the 40th anniversary of the journal, the current Editor-in-Chiefs have commissioned a special issue to celebrate this milestone in the journal’s history. In this editorial we survey the development of the journal and give an introduction to the special issue.The history of *Clinical & Experimental Metastasis* is intimately connected with that of the Metastasis Research Society (MRS). The journal was launched a year before the foundation of the MRS in 1984 by the founders of the society, and was immediately adopted as the official journal of the MRS [1, 5]. Throughout its forty-year existence, the journal has maintained close ties with the MRS, as reflected for example in the many papers published in the journal by members of the society, the involvement of society members in the editorial process, and support by the journal for the biennial conferences of the MRS. For many years MRS members received a complimentary copy of *Clinical & Experimental Metastasis* as part of their society subscription [4].

**Fig. 1:**
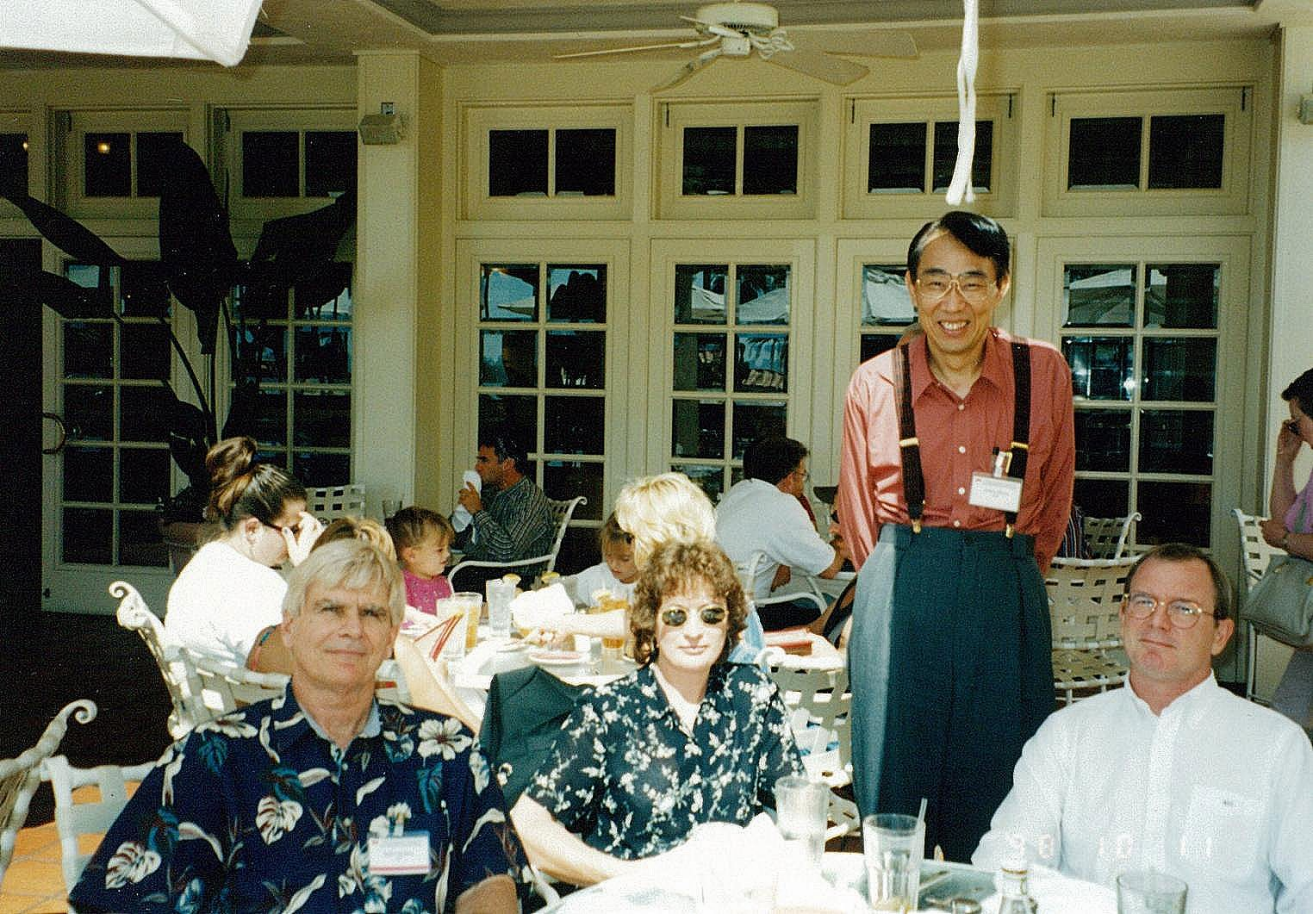
The *Clinical & Experimental Metastasis* editorial team in 1998. From left to right: Garth Nicolson (USA editor), Sue Eccles (European editor) and Tatsuro Irimura (editor for Asia and the far East), together with William Stetler-Stevenson (far right), the then president of the Metastasis Research Society. The photograph was taken at the 8th biennial conference of the Metastasis Research Society that was held in San Diego, USA in 1998. Photo courtesy of Tatsuro Irimura

The first editors of *Clinical & Experimental Metastasis* were Kurt Hellman, Garth Nicolson, and Sue Eccles, with Luca Milas acting as associate editor [1]. These editors were later joined by Tatsuro Irimura [2]. At that time editorial responsibilities were divided along geographical lines, with Garth Nicolson editing submissions from the US, Sue Eccles handling submissions from Europe and Tatsuro Irimura being responsible for submissions from Asia and the far East (Fig. 1). Kurt Hellman and Luca Milas retired from editorial duties in 1998, although Kurt Hellman continued to be involved in the journal as editor emeritus for some years afterwards [1]. Garth Nicolson left the editorial team in 2000, and his role as editor-in-chief for the USA was taken over by Danny Welsh [2].

The 25th anniversary issue of *Clinical & Experimental Metastasis* in 2009 was marked by the remaining editors (Sue Eccles, Tatsuro Irimura and Danny Welch) in an editorial in which they celebrated the successes of the journal over the previous quarter of a century [3]. Notable milestones included moving from four issues per year at its inception, to 6 issues per year in 1993 [4] and subsequently to the current eight issues per year [3]. When Danny Welch moved on to other editorial responsibilities in 2010, Robin Anderson took over his role as Editor-in-Chief for the USA [2].

The year 2016 saw some significant changes in the editorial structure of *Clinical & Experimental Metastasis*. Sue Eccles retired from her role as Editor-in-Chief after an amazing 32 years of service to the journal [2, 5]. She was replaced by Jonathan Sleeman, one of the current editors-in-chief. The other editors Robin Anderson and Tatsuro Irimura also stepped down from their editorial roles at the end of that year [6]. At that time, the majority of manuscripts published in the journal reported experimental findings, which had been the case since the inception of the journal. In order to strengthen the clinical focus of the journal, Jörg Haier, the other current editor-in-chief, was appointed in 2017 [7]. Editorial duties for the journal were then reorganised, with Jonathan Sleeman being responsible for basic research submissions, and Jörg Haier handling clinical research manuscripts.

Over the years *Clinical & Experimental Meta*stasis has been produced by a number of publishing houses. The first volumes of the journal were published by Taylor & Francis, which remained the case until 1990. Rapid Communications and then Rapid Science published the journal from 1991 until 1998. Two issues of the journal were published Lippincott Raven in 1998, and then the journal was taken over by Kluwer who were the publishers from late 1998 until the middle of 2004. Springer then acquired the journal, and has remained the publishers until the present day. The editors would like to take this opportunity to thank Springer Nature for their continuous and continued support for the journal, and especially the various publishing editors who have been assigned to the journal over the years. Working in the background, these individuals have made many valued contributions to the success of the journal and to the editorial process. The editors would like to thank Ying Jia in particular, who was publishing editor until recently, and look forward to working with Christina Bigdeli-Issazadeh who has just taken over this role.

In this Special Issue to mark the 40th Anniverary of *Clinical & Experimental Metastasis*, the editors have aimed to commission articles that reflect both the history of the journal, and the close connection of the journal with the MRS. Accordingly, papers in the Special Issue have been contributed by former and current Editors-in-Chief [8–10] (Tatsuro Isimura, Garth Nicolson and Jonathan Sleeman) and well as by former presidents of the MRS [10–12] (Jonathan Sleeman, Patricia Steeg and Dihua Yu). To reflect the mission of the MRS to promote the careers of young researchers, manuscripts have also been commissioned from former Chairs of the Early Career Leadership Council [13, 14] (Thomas Cox and Adrian Wiegmans), a section of the MRS devoted to the interests of society members who are an early stage of their careers in metastasis research (https://metastasis-research.org/for-researchers/eclc/).

Looking forward beyond the first 40 years of *Clinical & Experimental Metastasis*, the editors are reminded of the old adage that “life begins at 40”. We remain as committed as always to publishing the best basic and clinical research findings in the field of metastasis, as was outlined in the editorial published by the founding editors in the first issue of *Clinical & Experimental Metastasis* in 1983 [15]. The field of metastasis research has exploded dramatically since the inception of the journal, particularly during the last couple of decades, and we are proud of the role that the journal has played in providing a platform for supporting the rapid progress in this field over recent years. The publishing landscape has become increasingly crowded in this area, with many old and new journals competing for the best research. With this in mind the editors-in-chief would warmly encourage you as readers to consider *Clinical & Experimental Metastasis* as the journal to which you submit your next research findings. We look forward to working with you to increase the impact and scope of the journal in years to come. Onwards and upwards.

Jonathan Sleeman and Jörg Haier

Editors-in-chief, *Clinical & Experimental Metastasis*
